# Evaluation of the Quality and Educational Value of YouTube Videos on Class IV Resin Composite Restorations

**DOI:** 10.3390/dj13070298

**Published:** 2025-06-30

**Authors:** Rashed A. AlSahafi, Hesham A. Alhazmi, Israa Alkhalifah, Danah Albuhmdouh, Malik J. Farraj, Abdullah Alhussein, Abdulrahman A. Balhaddad

**Affiliations:** 1 Department of Restorative Dental Sciences, College of Dentistry, Umm Al-Qura University, Makkah 24211, Saudi Arabia; rasahafi@uqu.edu.sa; 2 Department of Preventive Dentistry, College of Dentistry, Umm Al-Qura University, Makkah 24211, Saudi Arabia; 3 Department of Oral Health Policy and Epidemiology, Harvard School of Dental Medicine, Boston, MA 02115, USA; 4 College of Dentistry, Imam Abdulrahman Bin Faisal University, Dammam 31441, Saudi Arabia; 5 Department of Restorative Dental Sciences, College of Dentistry, Imam Abdulrahman Bin Faisal University, P.O. Box 1982, Dammam 31441, Saudi Arabia; mjfarraj@iau.edu.sa; 6 Department of Restorative Dental Science, College of Dentistry, King Saud University, P.O. Box 60169, Riyadh 11545, Saudi Arabia; aalhussein@ksu.edu.sa

**Keywords:** composite, education, dental, YouTube

## Abstract

**Objectives:** The increasing reliance on online platforms for dental education necessitates an assessment of the quality and reliability of available resources. This study aimed to evaluate YouTube videos as educational tools for Class IV resin composite restorations. **Methods**: The first 100 YouTube videos were screened, and 73 met the inclusion criteria. The videos were evaluated using the Video Information and Quality Index (VIQI) and specific content criteria derived from the dental literature. Videos with a score below the mean were identified as low-content videos. **Results**: No significant differences were noted between high- and low-content videos when examining the number of views, number of likes, duration, days since upload, viewing rate, interaction index, and number of subscribers (*p* > 0.05). The high-content videos demonstrated higher mean values compared with the low-content videos in flow (4.11 vs. 3.21; *p* < 0.0001), accuracy (4.07 vs. 3.07; *p* < 0.0001), quality (4 vs. 2.66; *p* < 0.0001), and precision (4.16 vs. 2.86; *p* < 0.0001). The overall VIQI score was significantly higher (*p* < 0.0001) for high-content videos (Mean 16.34; SD 2.46) compared with low-content videos (Mean 11.79; SD 2.96). For content score, high-content videos (Mean 9.36; SD 1.33) had a higher score (*p* < 0.0001) than low-content videos (Mean 4.90; SD 2.04). The key areas lacking sufficient coverage included occlusion, shade selection, and light curing techniques. **Conclusions**: While a significant portion of YouTube videos provided high-quality educational content, notable deficiencies were identified. This analysis serves as a call to action for both content creators and educational institutions to prioritize the accuracy and completeness of online dental education.

## 1. Introduction

For nearly two centuries, there has been a growing need for tooth-colored restorations, cosmetic dental procedures, and the preservation of tooth structure [1,2]. Consequently, the placement of direct composite restorations has become widespread [3,4]. The success of direct composite restorations is closely correlated with operator techniques, operator clinical skills, material understanding, and advances in adhesive technology [5,6,7]. In most restorative practices, direct composite restoration placement is a fundamental procedure, and recent studies emphasize the necessity for comprehensive training in direct composite restoration procedures, particularly in complex clinical scenarios such as Class IV and aesthetic anterior restorations [8].

E-learning, also known as electronic or online learning, has emerged as an innovative and practical teaching approach [9]. It leverages electronic technologies to facilitate knowledge attainment beyond the boundaries of traditional classroom education. Online platforms such as YouTube™ are commonly used to access a vast amount of information [10]. One of the most important privileges of this type of platform is the graphic illustration of practical techniques [11,12]. Many students in the medical and dental fields utilize YouTube™ as an instructive resource to train for practical techniques, even though its main purpose is not educational [13]. Moreover, numerous reports have shown that YouTube™ is one of the resources that is most commonly used by healthcare students [13].

In addition to the growing dependence on digital media for educational resources, the rapid advancement of dental materials and clinical procedures further emphasizes the significance of continuous learning in dentistry [14]. Newer generations of composite restorations demonstrate improved aesthetic and mechanical properties, easier handling techniques, and better long-term success [14]. These advancements enable them to closely mimic the aesthetic characteristics of enamel and dentin, resulting in highly aesthetic outcomes, especially in Class IV cavity preparation.

Class IV cavity preparation is used to restore anterior teeth that have suffered from caries or trauma, particularly affecting the incisal edge and interproximal surfaces [15,16,17]. Restoring Class IV cavities can be challenging due to their complex anatomy, which requires precise shaping and contouring, as well as effective bonding to both enamel and dentin in areas subject to significant functional and aesthetic demands. Additionally, achieving a seamless blend with the surrounding tooth structure while ensuring durability can complicate the restoration process [15,16,17]. Students tend to learn about different techniques and advancements in restoring Class IV cavity preparation from accessible educational sources.

Nowadays, with the increased number of online educational platforms, it is crucial for students to have basic evaluation skills when searching for information online [18]. By establishing these measures, dental students can gain the ability to distinguish between high- and low-quality educational videos, which will enrich their academic knowledge and clinical skills with accurate and current information. YouTube™ allows for users to post videos with no proof of their knowledge or expertise [19,20]. As a result, there is no assurance that the data shared in the videos are accurate or current. Students may struggle to evaluate the reliability of the content presented [20,21,22].

To address this, a comprehensive assessment of accessible YouTube™ videos on Class IV resin composite restorations is crucial. This evaluation will help determine whether these videos can serve as effective teaching tools. It is important to highlight the significant lack of research addressing the evaluation of online platforms concerning aesthetic direct composite restorations. Therefore, this study assessed the quality and content of YouTube™ videos on restoring Class IV cavity preparations.

## 2. Materials and Methods

### 2.1. Sample Size and Search Strategy

Previous reports have shown that most research papers focusing on YouTube™ analysis include the first one hundred videos shown in the search results [18]. These videos are highly accessible to users. Therefore, our investigation used a sample size of 100 videos. Additionally, we investigated frequently searched terms by analyzing their relative search volume across different global regions using the Google Trends tool; in particular, we examined whether the term ‘Class IV composite restoration’ was the most used term. To ensure a comprehensive and unbiased search, we accessed Google Trends data from the past 5 years, with both ‘worldwide’ and ‘secret’ settings enabled.

YouTube™ (https://www.YouTube.com, accessed on 10 October 2024), a popular online video sharing platform, primarily depends on a “relevance level” filter to sort search outcomes. In this study, a search was conducted to evaluate materials correlated with Class IV resin composite restorations. The evaluation focused on the first 100 videos, excluding commercial advertisement videos. The search results were compiled in a list, and the video URLs were securely backed up.

### 2.2. Video Analysis and Evaluation

YouTube™ videos were screened by two independent reviewers. To ensure inter-rater reliability, a third reviewer was added. The exclusion criteria involved non-English videos, videos without sound, and duplicated videos. Where videos had more than one part, the parts were treated as a single video. All data associated with the video were documented, including the type of account, the duration of content in minutes, the number of views/likes/subscribers, viewing rate, interaction index, upload date, tooth number, and type of patents. The following criteria were considered during the evaluation of the YouTube™ videos:

(1) YouTube™ videos were classified into three categories: educational, layperson, and commercial, which include supply companies or dental manufacturing.
(2) The overall quality of the videos was evaluated using the ‘Video Information and Quality Index’ (VIQI) (Table 1). The VIQI index uses a 5-point rating scale, ranging from high quality (5) to poor (1), to evaluate different aspects of the videos. These aspects involve quality, accuracy, and information flow. One point is awarded for interviews with experts in the field, use of animation, a summary report, and video captions. Additionally, precision is evaluated by evaluating the coherence between the content and the video title.
(3) In addition to the VIQI score, the quality of the YouTube™ videos was evaluated using specific standards associated with Class IV composite restorations. These criteria, which are detailed in Table 2, are derived from the textbook *Modern Operative Dentistry* [23]. The total scoring system comprises 13 points, with each criterion contributing 1 point. The average score was computed to assess the content value of the reviewed videos. Videos that met or exceeded the average score were classified as high-content, whereas those falling below the average were labeled as low-content.
(4) In this study, the educational value of each video was defined as a combined measure that merged both the quality of instructional delivery and technical accuracy. Instructional quality was evaluated using the Video Information and Quality Index (VIQI) as outlined in Table 1. In addition, technical accuracy was assessed using specific content criteria derived from *Modern Operative Dentistry* (Table 2), encompassing key procedural steps including isolation, tooth preparation, and restorative techniques. Videos that demonstrated high performance in both technical and instructional aspects were considered to have high educational value.
(5) The level of viewer interaction with the videos was assessed using a specific formula to calculate the viewing rate. This method entails dividing the total number of views by the number of days since the video was uploaded and multiplying that quotient by 100 [10].

**Table 1:** Parameters of the Video Information and Quality Index (VIQI) and their corresponding scores utilized in this study.

Aspect	1 Point	2 Points	3 Points	4 Points	5 Points
Flow of information	Disjointed; difficult to follow	Partially disorganized; flow needs improvement	Reasonably organized; some flow issues	Well-organized; easy to follow	Seamless flow; logically structured and engaging
Accuracy	Frequent inaccuracies; misleading information	Some inaccuracies; questionable content	Generally accurate; minor inaccuracies	Mostly accurate; reliable information	Highly accurate; thoroughly verified.
Quality	Poor production; distracting elements	Low quality; noticeable issues present	Average quality; some minor distractions	Good quality; few distractions	Excellent quality; professionally produced
Precision	Extremely vague; no relevant details	Some relevant details, but mostly unclear	Moderately clear; some useful details	Generally clear; most details relevant	Highly precise; all details relevant and clear

**Table 2:** A list of the criteria used to evaluate the content of Class IV resin composite restoration videos on YouTube and to estimate the final content score. Each criterion represents one point, resulting in a total of 13 points. The criteria were extracted from the textbook *Modern Operative Dentistry*.

#	Criteria	=1	=0
1	Isolation	Demonstrate proper isolation method used. Proper isolation method is defined as complete tooth isolation using a rubber dam, with appropriate clamp selection and a secure seal.	Did not demonstrate proper isolation or used incorrect method.
2	Tooth preparation	Demonstrate accurate tooth preparation guidelines based on *Modern Operative Dentistry* (proximal clearance, removal of unsupported enamel, no remaining caries, occlusal wall convergence, 90-degree margins).	Did not demonstrate accurate tooth preparation guidelines.
3	Bevels	Demonstrate correct bevel application.	Did not demonstrate correct bevel application, or no bevel used.
4	Bur used	Demonstrate a clear rationale for bur selection during tooth preparation, specifying the type and shape used at each step.	Did not demonstrate appropriate bur selection or none used.
5	Matrix application	Demonstrate correct matrix applied for restoration.	Did not demonstrate correct matrix application or none used.
6	Wedge application	Demonstrate correct wedge direction for proper adaptation.	Did not demonstrate correct wedge direction or none used.
7	Adhesive placement technique used	Demonstrate proper adhesive technique.	Did not demonstrate proper adhesive technique or none used.
8	Composite insertion technique	Demonstrate correct composite insertion technique.	Did not demonstrate correct composite insertion technique.
9	Light curing angulation and time	Demonstrate correct curing time and angle for effective polymerization.	Did not demonstrate correct curing time or angle.
10	Finishing of resin composite	Demonstrate proper finishing tools used for optimal restoration.	Did not demonstrate proper finishing tools or none used.
11	Polishing of resin composite	Demonstrate correct polishing method for restoration.	Did not demonstrate correct polishing method or none performed.
12	Proximal contact	Demonstrate correct contact established for proximal surfaces.	Did not demonstrate correct contact establishment.
13	Occlusion	Demonstrate correct occlusion check and adjustment.	Did not demonstrate correct occlusion check or adjustment.

### 2.3. Statistical Analysis

Statistical analysis was conducted using SAS 9.4 (SAS Institute, Cary, NC, USA). Descriptive statistics (mean, frequencies, standard deviation, minimum, and maximum value) were calculated for all variables. The Kolmogorov–Smirnov and Shapiro–Wilk tests were used to ensure data normality. Student’s *t*-test was used to compare characteristic and quality parameters. Moreover, the chi-squared test or Fisher’s exact test was used to determine the differences in content elements and source between high- and low-content video categories. The significance level was set at *p* < 0.05.

## 3. Results

Among the screened videos (*n* = 100), seventy-three were included in the analysis. The excluded videos were non-English or unrelated to the topic. Table 3 contains the descriptive data for the videos included in this study. The mean number of views is 49,568.48 ± 194,481.80, while the mean number of likes is 942.64 ± 3665.56. Additionally, the mean duration of the videos is 37.56 min ± 96.10, and the mean day since upload is 1791.03 ± 1003.67. Furthermore, the mean viewing rate is 2159.92 ± 6232.02, and the mean interaction index is 40.70 ± 223.54. The mean number of subscribers is 55,599.45 ± 146,716.90.

Table 4 contains the mean and standard deviations for the VIQI and content scores. For the VIQI score, the mean flow score is 3.75 ± 0.91, while the mean accuracy score is 3.67 ± 0.97. Additionally, the mean quality score is 3.47 ± 1.13, and the mean precision score is 3.64 ± 1.05. The mean overall VIQI score is 14.53 ± 3.48. The mean content score is 7.59 ± 2.74. Based on the mean, videos were classified as high-content if they scored 7.59 or higher and low-content if they scored below 7.59.

Using the content score as a reference, Table 5 illustrates the characteristic variables of high-content (≥7.59) and low-content (<7.59) videos. The variables assessed included duration, number of subscribers, the number of likes, the number of views, viewing rate, days since upload, and interaction index. None of these variables had a significant *p*-value of less than 0.05, indicating no significant difference between high-quality and low-quality videos.

For video sources, the percentages of commercial, dentist, and layperson sources were assessed, with no significant *p*-value observed. For the type of account, the percentages of commercial, educational, and personal accounts were considered, with no significant *p*-value observed. None of these variables had a significant *p*-value (*p* < 0.05), as shown in Table 6.

When comparing the VIQI and content scores between high- and low-content videos, the mean flow for high-content videos was 4.11 ± 0.65, while low-content videos had a mean flow of 3.21 ± 0.98. For accuracy, high-content videos averaged 4.07 ± 0.82 compared with 3.07 ± 0.88 for low-content videos. The quality mean for high-content videos was 4.00 ± 0.86, whereas low-content videos averaged 2.66 ± 1.01. In terms of precision, high-content videos had a mean of 4.16 ± 0.81, while low-content videos scored 2.86 ± 0.88. The VIQI score for high-content videos was 16.34 ± 2.46, in contrast to 11.79 ± 2.96 for low-content videos. Lastly, the content score for high-content videos was 9.36 ± 1.33, compared to 4.90 ± 2.04 for low-content videos. All differences were statistically significant, with *p*-values less than 0.0001, as shown in Table 7.

**Figure 1:**
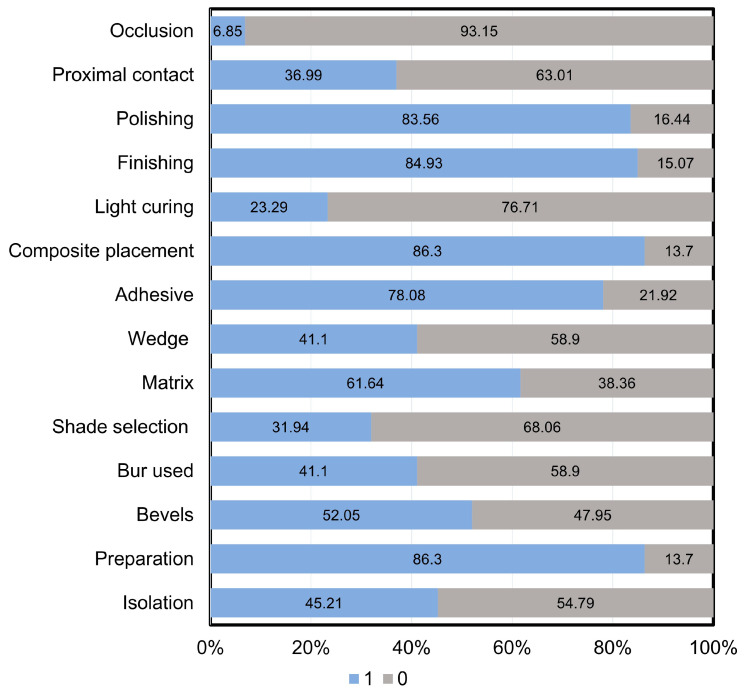
The percentages of the 13 criteria observed across the 73 videos. These criteria collectively contribute to a total score of 13 points, with each criterion assigned one point. A score of 1 indicates that the criterion is demonstrated in the video, while a score of 0 signifies that the criterion is missing.

**Figure 2:**
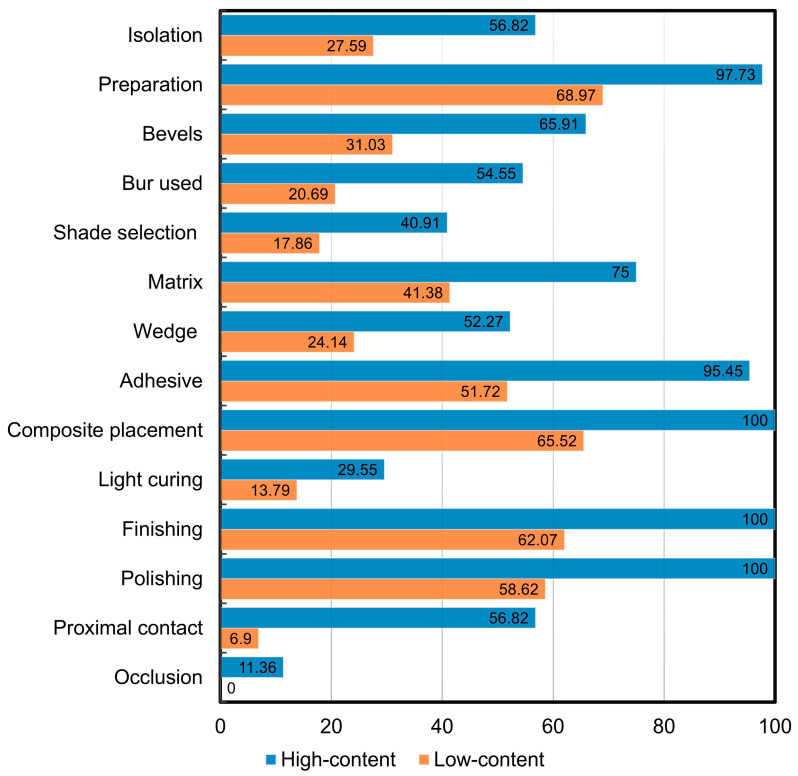
Percentages of the 13 criteria observed in high-content and low-content videos.

Figure 1 offers a comprehensive overview of how the criteria in the content score evaluation (see Table 2) are distributed among the 73 analyzed videos. The assessment revealed several areas lacking sufficient coverage, particularly in occlusion, shade selection, and the appropriate use of light-curing units. On the other hand, the criteria that were most showcased in the videos included tooth preparation, composite placement, and finishing and polishing processes. Figure 2 illustrates the shortcomings in the identified criteria by categorizing the reviewed videos into high-content and low-content groups. Notably, both high- and low-content videos exhibited significant weaknesses in occlusion, shade selection, and the implementation of light-curing units. Additionally, the low-content videos also demonstrated notable deficiencies in the restoration and contouring of proximal contacts.

## 4. Discussion

Many studies show a substantial expansion in the number of students using YouTube as an educational resource [24]. YouTube can enhance knowledge retention and increase student interest due to its dynamic, accessible, and easy-to-use nature [25,26]. These criteria make YouTube a popular platform for accessing educational and informational content. However, in the dental and medical education fields, more findings are necessary to validate the educational efficiency of YouTube videos. Due to the platform’s open nature, the reliability, validity, and accuracy of educational content are questionable [20]. There is no peer review process by which reviewers or specialists can verify, correct, or edit YouTube content before publication [27]. This study assessed the quality of YouTube videos that depict Class IV resin composite restorations. The findings revealed that most of the videos reviewed were categorized as high-content, with 44 videos falling into this group compared with 29 videos that were classified as low-content. Furthermore, the high-content videos exhibited significantly superior flow, accuracy, quality, and precision in comparison to their low-content counterparts.

Previous studies have shown that educational videos uploaded by specialists and educational institutions have higher completeness scores than those from other sources. However, the overall completeness score was low, despite the higher scores from the educational sources [28]. Moreover, in a recent study, [13] more than 90% of dental students were found to utilize YouTube to learn clinical procedures, with around 36% unsure about the reliability and accuracy of the videos they watched. Therefore, it is critical that professional institutions provide more educational videos to lower the risk of misinformation. Additionally, it is important to officially validate established platforms through accreditation procedures and to create proactive strategies for online platforms to ensure the creation of accurate educational videos.

In aesthetic restoration, direct Class IV resin composite restorations pose significant challenges for long-lasting results [29]. A previous study by Heintze et al. found that Class IV restorations have double the failure rate of Class III restorations [30]. Moreover, a systematic review found that the survival rate of Class IV direct anterior composite restorations can vary widely, with a survival rate as low as 28% and as high as 100% over a period ranging from 2 to 10 years [29]. This variation indicates the sensitivity of this procedure and how evidence-based treatment can improve the survival rate. Many reports have reviewed the risk factors associated with the failure of the restoration [31]. These include color mismatch, fracture, nonvital teeth, adhesive techniques, and retreatment risk. The main causes of failure were aesthetics and fracture of the incisal edge [31]. Therefore, due to the complexity and sensitivity of this procedure, many dental students tend to use online media to review and enhance their clinical experience.

Due to the inclusion of fundamental information on Class IV direct anterior composite restoration in this study, we obtained high average scores for the screened videos. Our results align with another analysis of educational videos on tooth avulsions on YouTube, [32] where a high prevalence of high-quality content was found. Conversely, other reports showed a frequency of low-quality videos on pediatric education [33], crown preparation [34], and dental implant procedures [35]. Moreover, the content and VIQI scores showed a relationship between the quality and completeness of the uploaded videos; that is, high-content videos exhibited significantly higher VIQI scores than low-content videos, with statistically significant differences in quality, accuracy, flow, and precision. However, there were no significant differences in video engagement views, likes, and duration of upload between high- and low-content videos. This finding demonstrates the importance of professional accreditation and improving the reliability and accuracy of the uploaded educational video content. Additionally, it highlights the essential impact of academic professors in steering their students to accurate online videos.

One potential reason for the higher number of reliable contents observed in most of the videos examined in this study is that individuals who do not have a dental background are generally less inclined to outline or showcase the clinical processes involved in dental procedures. A previous report found that, due to the complexity of the clinical and surgical techniques, most of these videos are often produced by professionals, which increases the likelihood of high-quality videos and reliable sources of information [36,37]. Dental students will continue to depend on online sources as a convenient reference for information, despite the present concerns about the quality and accuracy of the information. Positive interactions between students and teachers often drive effective learning; these interactions could help learners to be more engaged, receive direction based on their individual needs, and learn more efficiently. Previous studies have demonstrated that teachers who build good connections with their students can encourage learners to actively participate in their educational development due to a positive educational environment [38]. Unlike current classrooms, YouTube’s learning environment lacks these interactive components, limiting its potential for explanation, conversation, and direct supervision. Therefore, it is important for instructors to be aware of the information available on online platforms and to guide their dental students in reviewing the information they find to obtain accurate, current, and reliable information. This could help to minimize their acquisition of faulty information [39].

Using the VIQI and the comprehensive criteria-based evaluation for Class IV composite restorations could offer several benefits. The VIQI scoring system measures the quality of content subheadings separately and independently. On the other hand, the comprehensive criteria-based system focuses mainly on the completeness and technical accuracy of the restorative procedures. Combining the methods could help deliver a more comprehensive assessment by cross-verifying the quality from different angles, which helps increase the reliability of the evaluation, ensuring that both general video quality and specific procedural details are included. The comprehensive criteria-based evaluation ensures that the content follows the correct procedural and educational standards, and the VIQI scoring system evaluates the overall quality. This combination helps to ensure that high-quality videos maintain excellent quality and follow academic standards.

It is important to highlight that this study has certain limitations. Firstly, only the top 100 videos were investigated, which may introduce potential selection bias. YouTube’s algorithmic personalization may also influence the search results, further limiting the representativeness of the sample; consequently, it might not fully cover the wide range of educational content available on YouTube. Moreover, while the VIQI and comprehensive criteria-based evaluations aim for objectivity, there is an inherent level of subjectivity in this study; there are no standardized tools specifically designed to assess video-based resources. As a result, the video evaluations in this study were conducted subjectively by two individuals using a checklist created by the researchers, with the validity of the results confirmed by a third party. However, this subjective assessment is consistent with the typical approach used in many other publications in this field [21,33,37,40,41]. Finally, the dynamic nature of YouTube videos means that content is often removed or modified, possibly compromising this study’s reproducibility.

Although YouTube has the potential to play a significant role in dental education, its unregulated, user-generated nature raises ethical concerns regarding the reliability and accuracy of educational information. While this study evaluates the educational value of Class IV techniques using the VIQI and specific content criteria, the underlying information is based on a general academic consensus rather than formal peer review. Therefore, future measures should consider establishing guidelines or accreditation systems to supervise and verify the correctness of educational material on YouTube.

## 5. Conclusions

Most of the YouTube videos reviewed in this study were high-content—44 videos compared with 29 low-content videos. Moreover, the high-content videos had significantly greater accuracy, flow, quality, and precision than the low-content videos. The absence of standardized assessment tools and peer review brings into question the reliability and precision of the online content delivered; therefore, academic instructors play a vital role in guiding their students through YouTube resources, ensuring they build a foundation of accurate and dependable knowledge for their academic endeavors.

## Figures and Tables

**Table 3:** Descriptive statistics of the characteristics of the sampled YouTube videos.

Variable	Minimum	Maximum	Mean	SD
Number of Views	7	1,539,671	49,568.48	194,481.80
Number of Likes	0	29,000	942.6438	3665.56
Duration (min)	0.18	568	37.56	96.10
Days Since Upload	93	4492	1791.03	1003.67
Viewing Rate	0.63	34,660.77	2159.92	6232.02
Interaction Index	0	1504.04	40.70	223.54
Number of Subscribers	0	704,000	55,599.45	146,716.90

**Table 4:** Descriptive statistics of the quality parameters and the overall quality of the sampled YouTube videos.

Variable	Minimum	Maximum	Mean	SD
Flow	1	5	3.75	0.91
Accuracy	1	5	3.67	0.97
Quality	1	5	3.47	1.13
Precision	1	5	3.64	1.05
VIQI score	4	20	14.53	3.48
Content Score	1	12	7.59	2.74

**Table 5:** Comparison of the characteristic variables between the high-content and low-content YouTube videos.

Variable	High-Content Videos (*n* = 44)		Low-Content Videos (*n* = 29)		*p*-Value
	Mean	SD	Mean	SD	
Number of Views	73,549.96	248,001.10	13,182.79	23,047.51	0.20
Number of Likes	1382.30	4664.04	275.59	615.74	0.21
Duration (min)	44.49	116.07	27.05	53.56	0.45
Days Since Upload	1887.36	1080.93	1644.86	871.37	0.32
Viewing Rate	2995.11	7842.89	892.76	1626.99	0.16
Interaction Index	65.63	286.46	2.88	4.27	0.24
Number of Subscribers	65,867.17	159,377.50	40,374.90	126,805.50	0.47

**Table 6:** Comparison of the source and content of the high- and low-content YouTube videos.

Variable	High-Content Videos (*n* = 44)	Low-Content Videos (*n* = 29)	*p*-Value
Video source			
Commercial	15.91% (7)	17.24% (5)	0.584
Dentist	72.73% (32)	79.31% (23)	
Layperson	11.36% (5)	3.45% (1)	
Type of account			
Commercial	11.36% (5)	10.34% (3)	0.991
Educational	75.00% (33)	75.86% (22)	
Personal	13.64% (6)	13.79% (4)	

**Table 7:** Comparison of the quality parameters and total content between the high-content and low-content YouTube videos.

Variable	High-Content Videos (n = 44)		Low-Content Videos (n = 29)		*p*-Value
	Mean	SD	Mean	SD	
Flow	4.11	0.65	3.21	0.98	<0.0001
Accuracy	4.07	0.82	3.07	0.88	<0.0001
Quality	4	0.86	2.66	1.01	<0.0001
Precision	4.16	0.81	2.86	0.88	<0.0001
VIQI score	16.34	2.46	11.79	2.96	<0.0001
Content Score	9.36	1.33	4.90	2.04	<0.0001

## Data Availability

The data supporting this study’s findings are available from the corresponding author upon reasonable request.
